# The diagnostic journey of patients being investigated for myopathy in a tertiary centre in England

**DOI:** 10.1007/s00415-024-12737-y

**Published:** 2024-12-12

**Authors:** Zekai Qiang, Laura Barnett, Georgia Bingham, Oscar Han, Annabel Walsh, Martin Conwill, Harry E. McDonough, Christopher J. McDermott, Pamela J. Shaw, James J. P. Alix

**Affiliations:** 1 Sheffield Institute for Translational Neuroscience, Division of Neuroscience, University of Sheffield, Sheffield, UK; 2 Department of Clinical Neurophysiology, Royal Hallamshire Hospital, Sheffield Teaching Hospitals NHS Foundation Trust, Sheffield, UK; 3 Department of Neurology, Royal Hallamshire Hospital, Sheffield Teaching Hospitals NHS Foundation Trust, Sheffield, UK; 4 Cross-Faculty Neuroscience Institute, University of Sheffield, Sheffield, UK

**Keywords:** Myopathy, Diagnosis, EMG, Biopsy, Muscle

## Abstract

Myopathies are heterogenous and can provide a diagnostic puzzle. Many patients investigated for myopathy will go on to other diagnoses. An overall understanding of how patients are investigated for suspected myopathy is lacking. Our aim was to understand how patients were investigated for myopathy in our tertiary centre and the timeline of their diagnostic journey. Through local database searches over a 5-year period (2015–2019), we identified a final total of 770 patients investigated for myopathy. Of these, 29.7% went on to a diagnosis of myopathy. The top non-myopathy diagnoses were neuropathy, spinal pathology and ataxia. Both the myopathy and non-myopathy groups had symptoms for an extended period before reaching specialist services (both groups 104 weeks). Following a first hospital visit, median time to diagnosis was not significantly different (myopathy 46.9 weeks, non-myopathy 40.7 weeks, *p* > 0.05). Data on the diagnostic journey for specific myopathies was also collected, with inflammatory myopathies diagnosed most quickly and muscular dystrophies most slowly. Muscle MRI and biopsy had the best positive predictive values (82.7% and 83.1%, respectively), while EMG had the best negative predictive value (89.3%). A combination of CK, EMG and neuroaxis MRI (brain and spinal cord) yielded at least one correct test result with respect to final diagnosis in 98.9% of cases. In conclusion, patients in whom a muscle disease is considered experience significant diagnostic delay. The first step in the diagnostic journey should be able to identify both myopathy and non-myopathy cases.

**Supplementary Information:**

The online version contains supplementary material available at 10.1007/s00415-024-12737-y.

## Introduction

Myopathies are heterogeneous and can be difficult to diagnose. Disease progression may be slow, and patients may present with non-specific symptoms. While individual conditions are rare, UK data suggests the group as a whole has a prevalence of approximately 80 per 100,000 people, with the most common types of myopathy reported to be inflammatory myopathies (25 per 100,000) and muscular dystrophies (29.5 per 100,000) [3]. Historically, it has been difficult to achieve specific diagnoses and many conditions have been considered untreatable. However, the precision of diagnostic genetics has rapidly improved and the development pipeline of potential treatments for many myopathies is extremely promising. As well as alleviating patient uncertainty earlier, clinical trials and new treatments are more likely to be successful if patients can be diagnosed earlier in the disease course.

Data on the time to diagnosis and the diagnostic journey of patients investigated for myopathy are limited. Most reports focus on specific conditions. For example, a study of adult-onset congenital myopathies revealed an average diagnostic delay of around 5 years [19]. A systematic review on idiopathic inflammatory myopathies calculated a mean diagnostic delay of just over 2 years [17]. More general studies in the diagnostic pathway of patients investigated for myopathy are scare. One such example is a study from Germany, which considered the time to diagnosis in patients diagnosed with a range of different myopathies via a self-reported questionnaire, identifying a long time to diagnosis from the point of first healthcare contact (4.3 years) [24]. In paediatric neurology, studies in Duchenne muscular dystrophy appear to show an encouraging decrease in the time to diagnosis from over 2 years [5, 27], to less than 1 year [10].

Understanding the diagnostic journey of patients investigated for suspected myopathy can help improve diagnostic pathways, recruitment into clinical trials and, ultimately, result in better patient care. A number of patients will also be investigated for possible myopathy but go on to other diagnoses. Understanding how often this is the case, which conditions these patients are diagnosed with and how they are investigated can also help better improve resource utilisation.

Thus, the aim of this work was to understand the diagnostic journey of patients being investigated for suspected myopathy.

## Methods

### Data collection

The project was registered as a service evaluation within Sheffield Teaching Hospitals NHS Foundation Trust (reference number 11314). To collect data on all adult patients investigated for myopathy (not just patients with a final diagnosis of myopathy), we chose to search our local databases for key investigations: neurophysiology (electromyography, EMG), radiology (muscle MRI), genetics and pathology (muscle biopsy). We set an investigation window of 5 inclusive years: 2015–2019. This was done to ensure sufficient time to allow a diagnosis to be made (given the published data of diagnostic delay)^7^. All patients undergoing tests which raised the possibility of myopathy were included. This was done by searching for ‘myopathy’, ‘muscle disease’, ‘myositis’ and ‘myogenic’ within the request form and issued report. For each patient, we reviewed case notes and other investigation databases to collect the final diagnosis, demographic details (age, gender), speciality of referring clinician (neurology—general, neurology—neuromuscular, rheumatology, other), presenting symptoms, duration of symptoms, creatine kinase (considered normal/abnormal according to the European Federation of the Neurological Societies guidelines [12]), myositis antibody panel, EMG, radiological and pathological tests. When collecting final diagnoses in the myopathy group, ‘unspecified’ was given if the biopsy found only non-specific myopathic features and clinical observations/other tests did not reveal a specific aetiology.

### Specific aims and objectives

Our aim was to understand how patients have been investigated for myopathy in our tertiary centre. Our objectives were, for both patients with and without a final diagnosis of myopathy, to capture the duration of symptoms prior to first hospital contact, the test combinations used, the timeline of test utilisation and the time taken to reach a diagnosis.

### Data analysis

The demographic characteristics of the current cohort were summarised using descriptive statistics. Given the non-normal data distribution, the association between age and the final diagnosis was investigated using the Wilcoxon rank sum analysis. For categorical variables such as gender and the investigating specialty, Chi-squared tests were employed. The numbers and percentages were computed for each myopathy type and alternative diagnosis.

Diagnostic performance metrics, including sensitivity, specificity, positive predictive values, and negative predictive values, were calculated using standard definitions: Sensitivity = True Positives/(True Positives + False Negatives); Specificity = True Negatives/(True Negatives + False Positives); PPV = True Positives/(True Positives + False Positives); NPV = True Negatives/(True Negatives + False Negatives).

A test combination analysis was performed to determine the frequency of different sets of investigations. Frequencies for each investigation for the whole cohort and myopathy/non-myopathy groups were then calculated. For test combination performance, test results were reviewed and judged as to whether the result was correct relative to the final diagnosis. This provides added information from the sensitivity/specificity in myopathy analysis. For example, consider a non-myopathy case with final diagnosis of radiculopathy from imaging but a normal EMG. In the myopathy/non-myopathy analysis, EMG would be considered correct, but it could be considered to have missed a diagnosis it could, at least theoretically, have contributed to. The test combinations were considered using an OR operator i.e. from suite of three tests, only one would need to be correct with respect to diagnosis to be judged as a successful test combination. Finally, the average number of tests used for myopathy and non-myopathy patients were calculated and tested for statistical significance using Student’s *t*-test.

The Wilcoxon rank sum test was applied to evaluate differences in the time to investigations from initial referral for myopathy and non-myopathy groups. The Kruskal–Wallis test, followed by post-hoc Dunn’s test, was employed for multiple pairwise comparisons to assess the impact of myopathy subtypes and initial specialists seen on the time to diagnosis from symptom onset and from initial consultation. A Bonferroni correction was implemented to account for multiple testing. The Wilcoxon rank sum test was again applied to assess differences in time to diagnosis between myopathy and non-myopathy patients, using initial referral and symptom onset as starting points. Timelines were created, demonstrating the median duration in weeks from the initial hospital visit to the completion of different investigations and diagnosis. The duration of symptoms until the first hospital visit is described using median values. The first quartile, median, and third quartile values are calculated to describe the time taken for 25%, 50%, and 75% of patients to receive a diagnosis, respectively. All analyses were performed using RStudio Version 2023.09.0 + 463.

## Results

The initial search identified 928 patients. A total of 158 patients were excluded (e.g. due to being lost to follow-up, incomplete clinical information), leaving a final total of 770 patients. Of these, 229 had a final diagnosis of myopathy (29.7%). The median age of the total cohort was 56 years (Table 1). There was a balance of gender in the whole cohort, but males were significantly more likely to go on to a diagnosis of myopathy (56.3% of myopathy patients, *p* < 0.01). Most patients were investigated via non-neuromuscular neurology clinics (71%). The most common final myopathy diagnoses were inflammatory (24.9%), unspecified (15.3%) and muscular dystrophy (13.5%; Table 2). A wide range of different non-myopathy diagnoses were encountered, the top three were neuropathy (20.9%), spinal pathology (14.8%) and ataxia (11.3%; Table 2 and supplementary Table 1).

**Table 1:** Patient demographics and referring specialties

		Total *N* = 770	Myopathy *N* = 229	Non-myopathy *N* = 541	*P* value
Age Median (IQR)		56 (24)	59 (25)	54 (25)	0.02585, Wilcoxon rank sum
Gender	Male (%)	377 (49.0)	129 (56.3)	248 (45.8)	0.00979, Chi-squared
	Female (%)	393 (51.0)	100 (43.7)	293 (54.2)	
Specialist referral	Neuromuscular neurologist (%)	222 (28.8)	91 (39.7)	131 (24.2)	< 0.00001, Chi-squared
	Non-neuromuscular neurologist (%)	380 (49.4)	77 (33.6)	303 (56.0)	
	Rheumatologist (%)	120 (15.6)	48 (21.0)	72 (13.3)	
	Other (%)	48 (6.23)	13 (5.68)	35 (6.47)	

Patients with a final myopathy diagnosis were older and more likely to be male

**Table 2:** Subtypes of myopathy and non-myopathy diagnoses

Type of myopathy	*N *(%)
Inflammatory	57 (24.9)
Unspecified	35 (15.3)
Muscular dystrophy	31 (13.5)
IBM	30 (13.1)
Mitochondrial	21 (9.17)
Metabolic	12 (5.24)
Immune mediated	11 (4.80)
Toxic	11 (4.80)
Congenital	6 (2.62)
Neck extensor	5 (2.18)
Endocrine	3 (1.31)
Critical illness	3 (1.31)
Amyloid	1 (0.44)
Lysosomal	1 (0.44)
McLeod	1 (0.44)
Steroid-induced	1 (0.44)

Non-myopathy	*N* (%)
Neuropathy	113 (20.9)
Spinal pathology	80 (14.8)
Ataxia	61 (11.3)
Fibromyalgia	60 (11.1)
Functional neurological disorder	53 (9.80)
Musculoskeletal	39 (7.21)
Motor neurone disease	21 (3.88)
Chronic fatigue syndrome	13 (2.40)
Isolated hyperCKaemia	11 (2.03)
Movement disorders	11 (2.03)

**Fig. 1:**
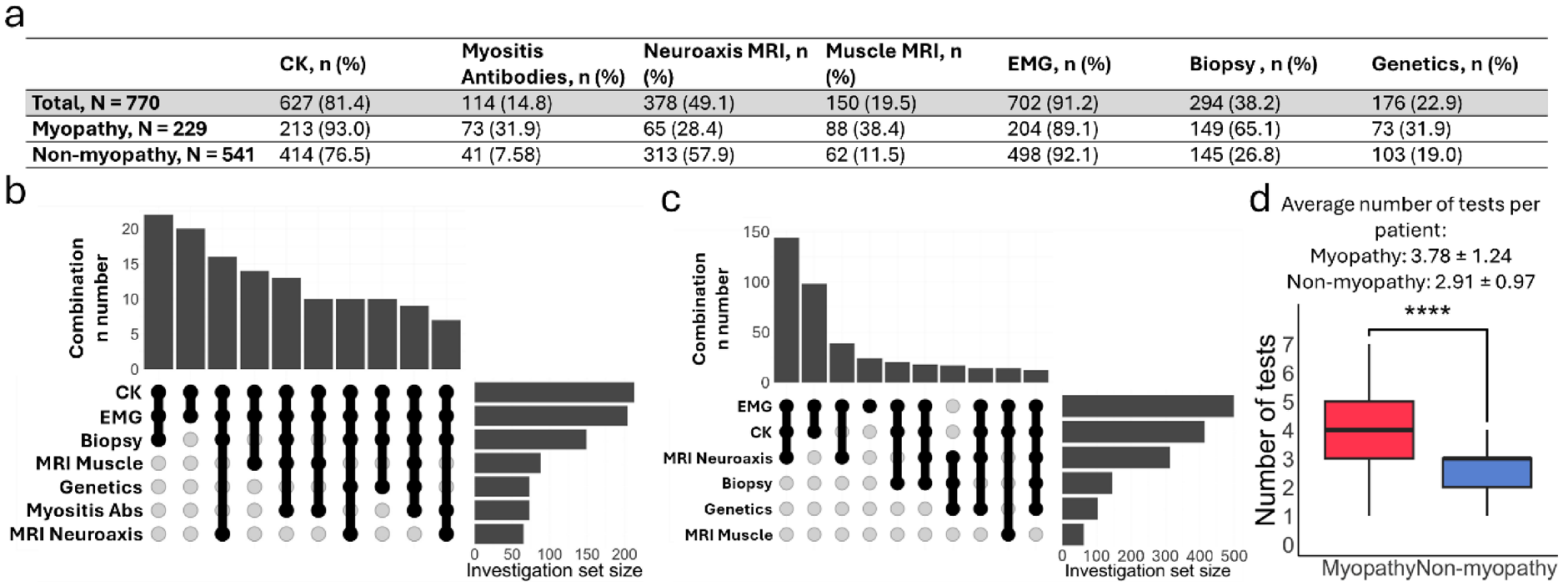
The utilisation of different tests for investigating possible myopathy. **a**. Use of the different investigations for the whole cohort and myopathy/non-myopathy diagnoses. CK and EMG were the most requested tests. **b**. Test combinations in the myopathy group. The most common combination of tests was CK + EMG + biopsy. **c**. Test combinations in the non-myopathy group. EMG + CK + MRI neuroaxis was the most frequently used combination. **d**. Patients with a final diagnosis of myopathy typically underwent more investigations

We next analysed the frequency and diagnostic performance of the tests used to identify patients under investigation for myopathy (EMG, muscle MRI, biopsy, genetics), as well as muscle-specific antibodies and MRI neuroaxis, since these were also often used in the diagnostic workup. EMG was the most frequently performed (91.2% in the total cohort, 89.1% of patients with a final diagnosis of myopathy and 92.1% of patients with a final non-myopathy diagnosis; Fig. 1a). The most frequent test combination in patients with a final diagnosis of myopathy was CK, EMG, and muscle biopsy (Fig. 1b). In patients with a final non-myopathy diagnosis, the top combination was EMG, CK and MRI neuroaxis (Fig. 1c). Myopathy patients underwent more tests than non-myopathy patients (*p* < 0.0001). Combinations used in specific diagnoses can be found in supplemental Tables 2–5 and supplementary Figs. 1–7.

The diagnostic performance of these investigations is shown in Table 3. Of note, myositis antibodies, biopsy and muscle MRI had the highest positive predictive values (92.6%, 83.1 and 82.7%, respectively), while the greatest negative predictive value was found in EMG (89.3%).

**Table 3:** Diagnostic performance of different tests in the assessment of myopathy

Investigation	*n*	Sensitivity (%)	Specificity (%)	PPV (%)	NPV (%)
CK	627	54.5	77.8	55.8	76.9
Myositis antibodies	114	34.3	95.1	92.6	44.8
EMG	702	73.5	90.6	76.1	89.3
Muscle MRI	150	70.5	79.0	82.7	65.3
Biopsy	294	72.5	84.8	83.1	75.0
Genetics	176	37.0	75.7	51.9	62.9

Note: we have not included MRI neuroaxis as, although this was commonly requested, the test was not looking for evidence of myopathy but rather to exclude structural pathologies

We also looked at the most informative combinations of tests in the cohort. If one test had to be correct with respect to the final diagnosis, the most successful combination was CK + EMG + MRI neuroaxis (Table 4).

**Table 4:** Successful combinations of tests

Combination	N All	N Correct	Percentage
CK + EMG	334	332	96.4
EMG + Neuroaxis MRI	265	250	94.3
CK + Neuroaxis MRI	184	175	95.1
**CK + EMG + Neuroaxis MRI**	**179**	**177**	**98.9**
EMG + Muscle Biopsy	110	101	91.8
CK + Muscle Biopsy	89	80	89.9
Neuroaxis MRI + Muscle Biopsy	80	75	93.8
CK + EMG + Muscle Biopsy	80	78	97.5
Genetics + EMG	67	64	95.5
Genetics + Muscle Biopsy	67	64	95.5

The most successful combination is shown in bold

**Fig. 2:**
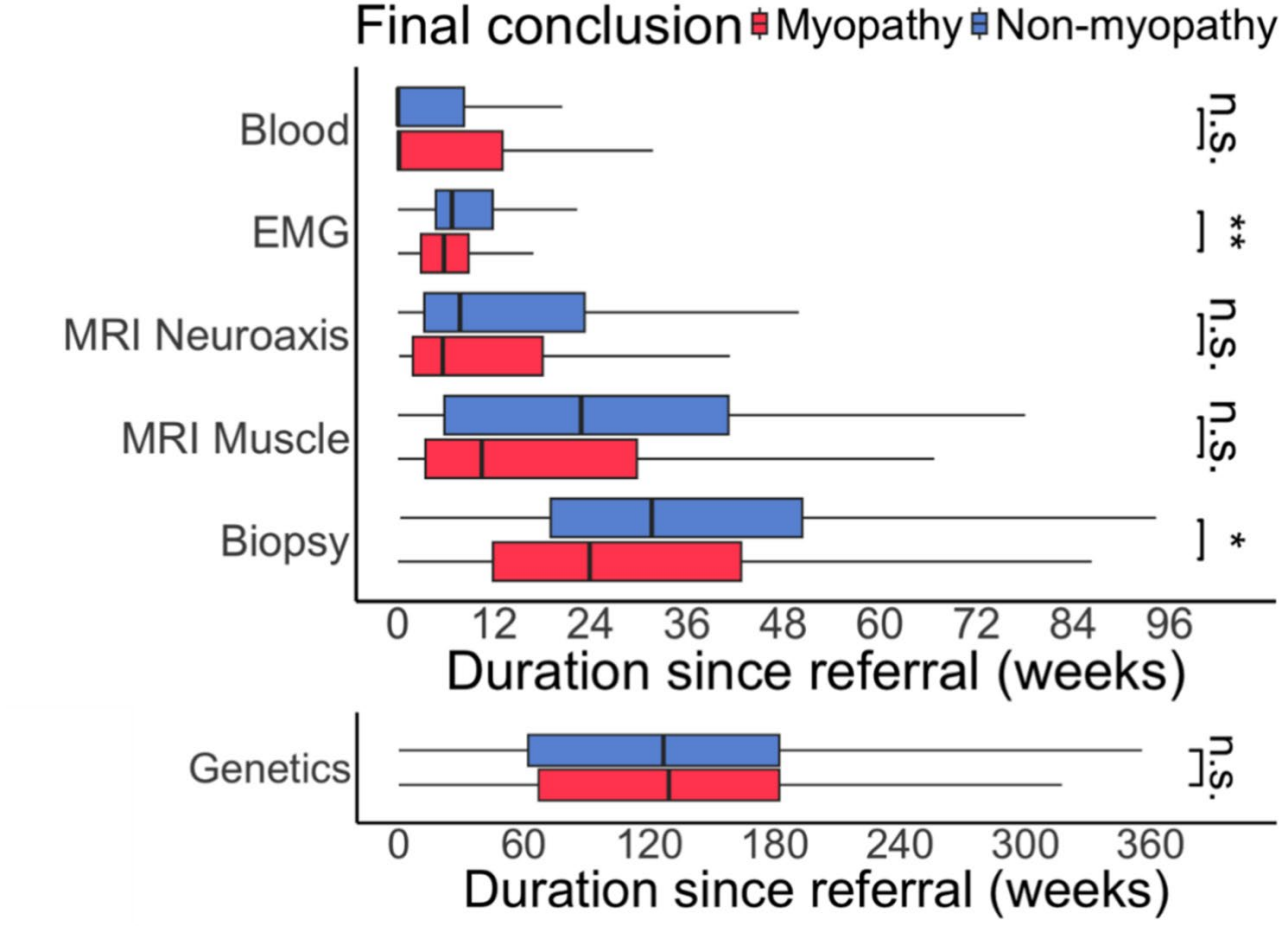
Variation in investigation timing. A wide variation in when tests were performed relative to the first hospital appointment was seen. However, patients with myopathy tended to undergo EMG and biopsy earlier than non-myopathy patients

We next looked at the time between the first hospital appointment, each of the investigations and diagnosis. There was marked variation in the time each test was performed (Fig. 2). There were differences evident between the myopathy/non-myopathy groups in EMG (median myopathy 5.7 weeks, non-myopathy 6.7 weeks, p < 0.01) and muscle biopsy (median myopathy 26.2 weeks, non-myopathy 34.6 weeks, *p* < 0.05).

**Fig. 3:**
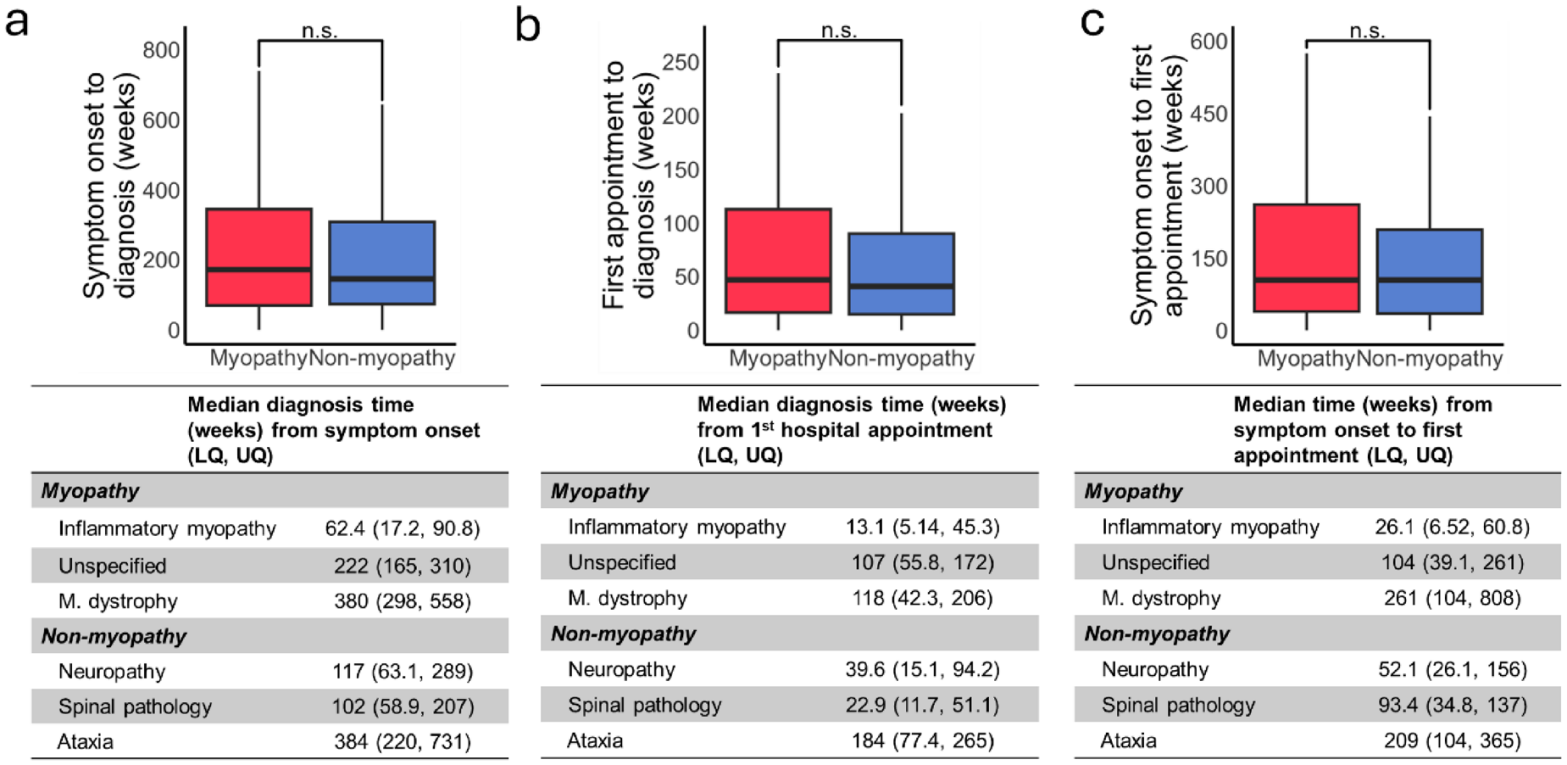
The times from symptom onset to diagnosis. **a**. Time from symptom onset to first hospital appointment was similar in both groups. The mean time for the top three diagnoses in each category is tabulated. **b**. The time from symptom onset to the final diagnosis was similar in both the groups. **c**. The time from first hospital appointment onset to the final diagnosis both from the first hospital consultant and symptom onset. *LQ* lower quartile, *UQ* upper quartile

The delay between symptom onset and first hospital consultation was the same in both groups (median 104 weeks) but varied considerably between different conditions (Fig. 3a). The time from symptom onset to diagnosis was also similar in both groups (Fig. 3b; median myopathy 171 weeks, non-myopathy 145 weeks, *p* > 0.05), as was the time from first hospital appointment to diagnosis (Fig. 3c; myopathy 46.9 weeks, non-myopathy 40.7 weeks, *p* > 0.05). Data for the most frequently encountered conditions can be found in Fig. 3a-c, the full lists for all conditions in both groups are contained within supplemental tables 5 and 6.

**Fig. 4:**
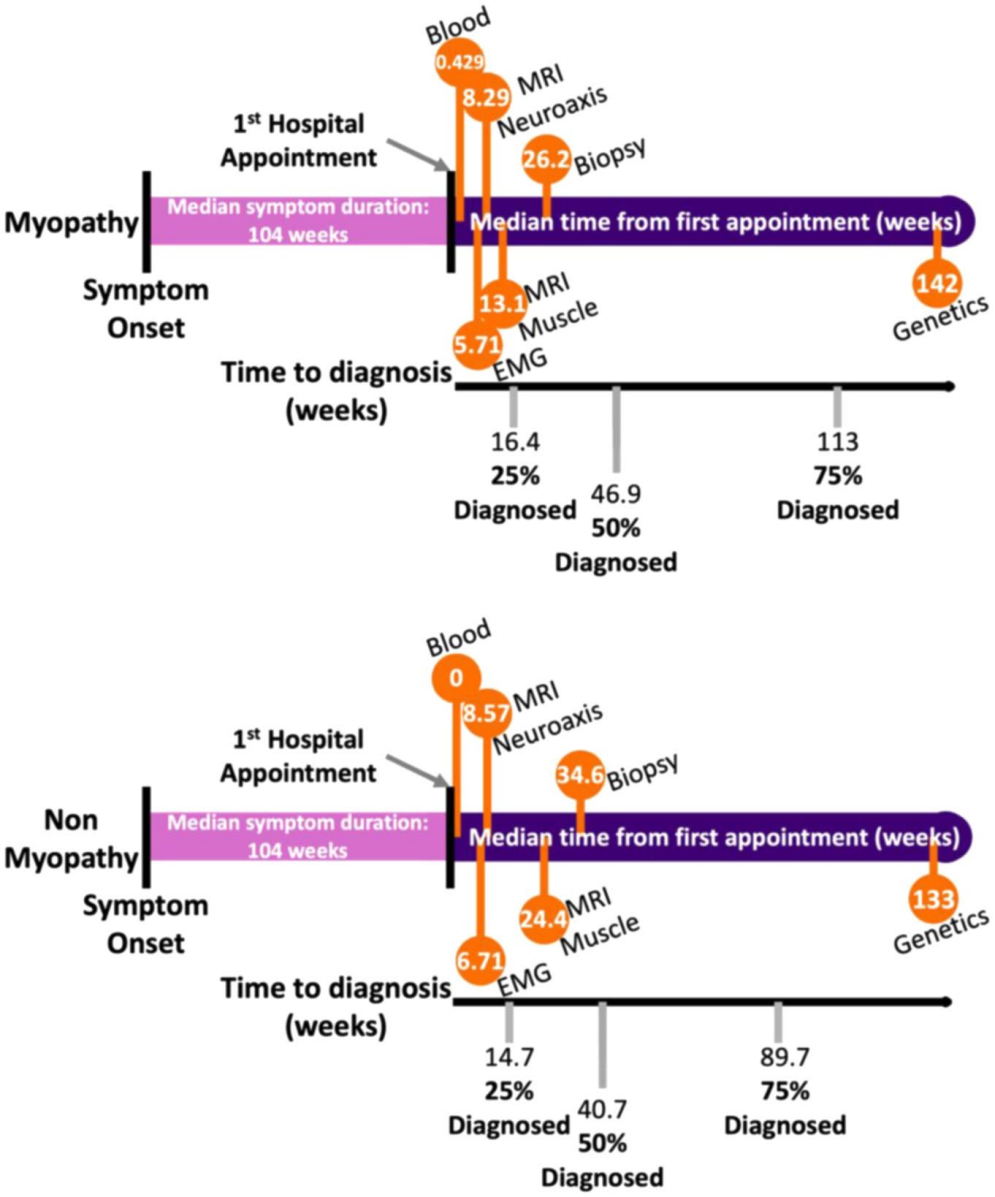
Overview of investigational and diagnostic timelines. Investigation timeline values are medians, diagnostic are inter-quartile range and median. All numbers shown are in weeks

Finally, representations of the diagnostic journey for myopathy and non-myopathy patients were calculated (Fig. 4). The diagnostic journey of cohort as a whole and the top three specific diagnoses within each category and the entire cohort (myopathy and non-myopathy) can be found in supplemental Figs. 2–7 and 8, respectively.

## Discussion

In this study, we have documented the diagnostic journey of patients being investigated for myopathy in our centre. We demonstrate that the majority of patients investigated for myopathy are found to have alternative diagnoses and that there is a considerable delay in both presentation to specialist services and a final diagnosis.

Both groups of patients experienced symptoms for a considerable time before reaching our centre. For the myopathy group, such a delay is not without precedent. For example, in Duchenne muscular dystrophy, UK data suggests 40 weeks may elapse before healthcare advice is sought [30]. However, most reports of myopathy diagnosis tend to focus on the overall diagnosis time (i.e. symptom onset and diagnosis e.g.[10, 17, 19, 24, 28]. Some authors have suggested that the long time to diagnosis in myopathy might be due to the relatively benign course of many myopathies [24], and/or the relatively non-specific nature of the symptoms [19]. In this regard, the shorter delay in presentation for inflammatory myopathies (26 weeks) likely relates to the more acute presentations within this cohort. In our data, the similar delay in the non-myopathy group would support the non-specific symptom hypothesis.

The overall time to diagnosis (symptom onset to diagnosis) in our myopathy cohort is highly varied, in keeping with prior literature on specific conditions. For example, reports on mitochondrial disease indicate diagnosis can take a mean of 10 years [28], for inflammatory myopathies, a systematic review reported a mean delay of 28 months [17], while IBM may take 5 years [6]. Our top non-myopathy diagnoses (neuropathy, spinal pathology, and ataxia) also often took considerable time to diagnose, in keeping with the literature for those conditions [4, 7, 11, 16]. Our data on the diagnostic performance of individual tests are in keeping with the prior literature [1, 2, 13–15, 18, 22, 23, 26, 29], and thus outlying diagnostic performance is not likely to be contributing to our results. Our data also suggest the primary use of some of these tests; for example, EMG may be most useful in excluding myopathy and identifying differential diagnoses. The value of muscle MRI is also demonstrated through the high positive predictive value, as reported in other studies [8, 25, 31].

What is clear is that a better diagnostic pathway is needed for this cohort of patients. The data indicate that a means of improving both the recognition of patients requiring specialist centre input (to reduce the time from symptom onset to hospital review) and a more streamlined diagnostic approach once in secondary/tertiary care (to reduce the time to diagnosis once in the specialist system) are required. Regarding the latter, pathways comprising initial tests that can effectively identify both myopathy and non-myopathy diagnoses appear to be of value. These can then, where necessary, inform further testing and help patients get to their underlying diagnosis as efficiently as possible. In muscle disease, this is particularly important if therapeutic developments are going to be leveraged effectively [6]. Our analysis suggests that a combination of CK, EMG and MRI neuroaxis has a high chance of moving forward towards both myopathy and non-myopathy diagnoses. By contrast, tests that only diagnose one side of the myopathy/not myopathy conundrum may fair less well, e.g. CK and biopsy (myopathy only) had the lowest percentage success rate (Table 4). Whether test combinations were done together, or sequentially, would depend upon the clinical and healthcare context and, of course, be for the clinician to decide. However, our data indicate that both myopathy and non-myopathy patients suffer diagnostic delay and thus early consideration of how to investigate both types of diagnosis may need to be given early consideration if timelines are to be reduced.

There are limitations to our investigation. As a single-centre study, the applicability of the findings to other centres and healthcare systems is uncertain. However, as noted above, the duration of symptoms, diagnostic performance of investigations, conditions encountered and time to diagnosis are similar to prior reports from a range of countries. There are no previous reports on large cohorts of patients in other healthcare systems, so perhaps our findings will stimulate others to look at their own diagnostic pathways. In doing so, the international neuromuscular community would be able to learn from systems that appear to provide a faster diagnostic approach, or reconsider how neuromuscular training is delivered in order to improve things for the future. Either way, our attempt to draw attention to the diagnostic odyssey of patients being investigated for myopathy has implications for the those working in neuromuscular neurology.

In order to identify patients diagnosed with myopathy and alternative conditions, we relied on database searches informed by core investigations. It is, therefore, possible that we will have missed patients in whom myopathy was considered but those investigations were not requested, e.g., we will have missed patients in whom a purely clinical diagnosis was made. We think that numbers of such patients are likely to be low. Our case note review did at least mean that we were not subject to the recall bias that may be encountered in patient surveys [9, 24]. We also did not collect data on whether one clinician started the diagnostic process and another clinician ended it, or where in the list of potential diagnoses myopathy fell (e.g. was it the primary consideration, or third or fourth on the list of differentials). Individual practice could also have influenced test choice and timing. Finally, our cohort contains a large number of ataxia diagnoses in the non-myopathy group. Our centre hosts a national ataxia unit and these patients were typically investigated with a biopsy looking for evidence of a mitochondrial cytopathy. In such cases, the muscle is effectively being used as a post-mitotic surrogate for the CNS and this practice may decline in the genomics first era of mitochondrial disease. As some of these patients did have evidence of mitochondrial myopathy of the biopsy and a final genetic diagnosis of mitochondrial disease, we retained this cohort within the study.

In conclusion, we found that patients investigated for suspected myopathy have symptoms for a long time before presenting to specialist services and then experience a long diagnostic delay, whether they are subsequently diagnosed with myopathy or not. We suggest that the first tests used in the diagnostic journey should have the ability to identify myopathy and its main differential diagnoses, as seen in our cohort. From our data, CK, EMG, and MRI neuroaxis represent a reasonable first diagnostic triage. These can be supplemented by genetics where clearly indicated (and they may also improve the yield [20, 21]) and then more invasive tests (e.g. biopsy). The development of more rapid diagnostic pathways will facilitate patient care and research.

## Supplementary Information

Below is the link to the electronic supplementary material.

Supplementary file1 (DOCX 3907 KB)

## Data Availability

Data are available upon request.

## References

[1] Bugiardini E, Morrow JM, Shah S, Wood CL, Lynch DS, Pitmann AM, Reilly MM, Houlden H, Matthews E, Parton M, Hanna MG, Straub V, Yousry TA (2018) The diagnostic value of MRI pattern recognition in distal myopathies. Front Neurol 9:456 29997562 10.3389/fneur.2018.00456 PMC6028608

[2] Cardy CM, Potter T (2007) The predictive value of creatine kinase, EMG and MRI in diagnosing muscle disease. Rheumatology (Oxford) 46:1617–1618 17704522 10.1093/rheumatology/kem211

[3] Carey IM, Banchoff E, Nirmalananthan N, Harris T, DeWilde S, Chaudhry UAR, Cook DG (2021) Prevalence and incidence of neuromuscular conditions in the UK between 2000 and 2019: a retrospective study using primary care data. PLoS ONE 16:e0261983 34972157 10.1371/journal.pone.0261983 PMC8719665

[4] Chaudhary UJ, Rajabally YA (2021) Underdiagnosis and diagnostic delay in chronic inflammatory demyelinating polyneuropathy. J Neurol 268:1366–1373 33170339 10.1007/s00415-020-10287-7 PMC7990867

[5] D’Amico A, Catteruccia M, Baranello G, Politano L, Govoni A, Previtali SC, Pane M, D’Angelo MG, Bruno C, Messina S, Ricci F, Pegoraro E, Pini A, Berardinelli A, Gorni K, Battini R, Vita G, Trucco F, Scutifero M, Petillo R, D’Ambrosio P, Ardissone A, Pasanisi B, Vita G, Mongini T, Moggio M, Comi GP, Mercuri E, Bertini E (2017) Diagnosis of duchenne muscular dystrophy in italy in the last decade: critical issues and areas for improvements. Neuromuscul Disord 27:447–451 28262469 10.1016/j.nmd.2017.02.006

[6] Dobloug GC, Antal EA, Sveberg L, Garen T, Bitter H, Stjärne J, Grøvle L, Gran JT, Molberg Ø (2015) High prevalence of inclusion body myositis in Norway; a population-based clinical epidemiology study. Eur J Neurol 22:672-e641 25530508 10.1111/ene.12627

[7] Gad H, Kalra S, Pinzon R, R-aN G, Yotsombut K, Coetzee A, Nafach J, Lim L-L, Fletcher PE, Lim V, Malik RA (2024) Earlier diagnosis of peripheral neuropathy in primary care: a call to action. J Peripher Nerv Syst 29:28–37 38268316 10.1111/jns.12613

[8] Gramegna LL, Rinaldi R, Belotti LMB, Vignatelli L, Sighinolfi G, Papa V, Costa R, D’Angelo R, Bianchini C, Graziano C, Cirignotta L, Mule R, Manners DN, Tonon C, Cenacchi G, Lodi R (2024) Magnetic resonance imaging scoring system of the lower limbs in adult patients with suspected idiopathic inflammatory myopathy. Neurol Sci. 10.1007/s10072-024-07386-y 38383748 10.1007/s10072-024-07386-y PMC11176218

[9] Grier J, Hirano M, Karaa A, Shepard E, Thompson JLP (2018) Diagnostic odyssey of patients with mitochondrial disease: Results of a survey. Neurol Genet 4:e230 29600276 10.1212/NXG.0000000000000230 PMC5873725

[10] Hiebeler M, Thiele S, Reilich P, Bernert G, Walter MC (2023) Time to diagnosis of Duchenne muscular dystrophy in Austria and Germany. Sci Rep 13:179 36604563 10.1038/s41598-022-27289-2 PMC9814243

[11] Indelicato E, Nachbauer W, Eigentler A, Amprosi M, Matteucci Gothe R, Giunti P, Mariotti C, Arpa J, Durr A, Klopstock T, Schöls L, Giordano I, Bürk K, Pandolfo M, Didszdun C, Schulz JB, Boesch S (2020) Onset features and time to diagnosis in Friedreich’s Ataxia. Orphanet J Rare Dis 15:198 32746884 10.1186/s13023-020-01475-9 PMC7397644

[12] Kyriakides T, Angelini C, Schaefer J, Sacconi S, Siciliano G, Vilchez JJ, Hilton-Jones D (2010) EFNS guidelines on the diagnostic approach to pauci- or asymptomatic hyperCKemia. Eur J Neurol 17:767–773 20402744 10.1111/j.1468-1331.2010.03012.x

[13] Lai CH, Melli G, Chang YJ, Skolasky RL, Corse AM, Wagner KR, Cornblath DR (2010) Open muscle biopsy in suspected myopathy: diagnostic yield and clinical utility. Eur J Neurol 17:136–142 19674068 10.1111/j.1468-1331.2009.02765.x

[14] Mammen AL, Casciola-Rosen L, Christopher-Stine L, Lloyd TE, Wagner KR (2015) Myositis-specific autoantibodies are specific for myositis compared to genetic muscle disease. Neurol Neuroimmunol Neuroinflamm 2:e172 26668818 10.1212/NXI.0000000000000172 PMC4676353

[15] Moloney PB, Lefter S, Ryan AM, Jansen M, Bermingham N, McNamara B (2021) The diagnostic yield of electromyography at detecting abnormalities on muscle biopsy: a single center experience. Neurodiagn J 61:86–94 34120582 10.1080/21646821.2021.1916730

[16] Munro CF, Yurac R, Moritz ZC, Fehlings MG, Rodrigues-Pinto R, Milligan J, Margetis K, Kotter MRN, Davies BM (2023) Targeting earlier diagnosis: what symptoms come first in degenerative cervical myelopathy? PLoS ONE 18:e0281856 37000805 10.1371/journal.pone.0281856 PMC10065274

[17] Namsrai T, Parkinson A, Chalmers A, Lowe C, Cook M, Phillips C, Desborough J (2022) Diagnostic delay of myositis: an integrated systematic review. Orphanet J Rare Dis 17:420 36411487 10.1186/s13023-022-02570-9 PMC9677896

[18] Ng KWP, Chin HL, Chin AXY, Goh DL (2022) Using gene panels in the diagnosis of neuromuscular disorders: a mini-review. Front Neurol 13:997551 36313509 10.3389/fneur.2022.997551 PMC9602396

[19] Nicolau S, Liewluck T, Tracy JA, Laughlin RS, Milone M (2019) Congenital myopathies in the adult neuromuscular clinic: Diagnostic challenges and pitfalls. Neurol Genet 5:e341 31321302 10.1212/NXG.0000000000000341 PMC6563518

[20] Nicolau S, Milone M, Liewluck T (2021) Guidelines for genetic testing of muscle and neuromuscular junction disorders. Muscle Nerve 64:255–269 34133031 10.1002/mus.27337

[21] Rosenberg A, Tian C, He H, Ulm E, Collins Ruff K, B. Nagaraj C, (2023) An evaluation of clinical presentation and genetic testing approaches for patients with neuromuscular disorders. Am J Med Genet A 191:2679–2692 37503964 10.1002/ajmg.a.63356

[22] Satoh M, Tanaka S, Ceribelli A, Calise SJ, Chan EK (2017) A Comprehensive Overview on Myositis-Specific Antibodies: New and Old Biomarkers in Idiopathic Inflammatory Myopathy. Clin Rev Allergy Immunol 52:1–19 26424665 10.1007/s12016-015-8510-y PMC5828023

[23] Shaibani A, Jabari D, Jabbour M, Arif C, Lee M, Rahbar MH (2015) Diagnostic outcome of muscle biopsy. Muscle Nerve 51:662–668 25187298 10.1002/mus.24447

[24] Spuler S, Stroux A, Kuschel F, Kuhlmey A, Kendel F (2011) Delay in diagnosis of muscle disorders depends on the subspecialty of the initially consulted physician. BMC Health Serv Res 11:91 21542919 10.1186/1472-6963-11-91 PMC3112398

[25] Tasca G, Monforte M, De Fino C, Kley RA, Ricci E, Mirabella M (2015) Magnetic resonance imaging pattern recognition in sporadic inclusion-body myositis. Muscle Nerve 52:956–962 25808807 10.1002/mus.24661

[26] ten Dam L, van der Kooi AJ, van Wattingen M, de Haan RJ, de Visser M (2012) Reliability and accuracy of skeletal muscle imaging in limb-girdle muscular dystrophies. Neurology 79:1716–1723 23035061 10.1212/WNL.0b013e31826e9b73

[27] Thomas S, Conway KM, Fapo O, Street N, Mathews KD, Mann JR, Romitti PA, Soim A, Westfield C, Fox DJ, Ciafaloni E, for the Muscular Dystrophy Surveillance T, Network R (2022) Time to diagnosis of Duchenne muscular dystrophy remains unchanged: Findings from the Muscular Dystrophy Surveillance, Tracking, and Research Network, 2000–2015. Muscle & Nerve 66:193–197 35312090 10.1002/mus.27532 PMC9308714

[28] Thompson JLP, Karaa A, Pham H, Yeske P, Krischer J, Xiao Y, Long Y, Kramer A, Dimmock D, Holbert A, Gorski C, Engelstad KM, Buchsbaum R, Rosales XQ, Hirano M (2023) The evolution of the mitochondrial disease diagnostic odyssey. Orphanet J Rare Dis 18:157 37349818 10.1186/s13023-023-02754-x PMC10288668

[29] Van De Vlekkert J, Maas M, Hoogendijk JE, De Visser M, Van Schaik IN (2015) Combining MRI and muscle biopsy improves diagnostic accuracy in subacute-onset idiopathic inflammatory myopathy. Muscle Nerve 51:253–258 24895239 10.1002/mus.24307

[30] van Ruiten HJ, Straub V, Bushby K, Guglieri M (2014) Improving recognition of Duchenne muscular dystrophy: a retrospective case note review. Arch Dis Child 99:1074–1077 25187493 10.1136/archdischild-2014-306366 PMC4251173

[31] Verdú-Díaz J, Alonso-Pérez J, Nuñez-Peralta C, Tasca G, Vissing J, Straub V, Fernández-Torrón R, Llauger J, Illa I, Díaz-Manera J (2020) Accuracy of a machine learning muscle MRI-based tool for the diagnosis of muscular dystrophies. Neurology 94:e1094–e1102 32029545 10.1212/WNL.0000000000009068

