# Supplementary material for: The diagnostic journey of patients being investigated for myopathy in a tertiary centre in England
Source: J Neurol. 2024 Dec 12;272(1):35. doi: 10.1007/s00415-024-12737-y (PMC11638302; doi:10.1007/s00415-024-12737-y)
Supplement: Supplementary file 1 — Supplementary file1 (DOCX 3907 KB) [file 415_2024_12737_MOESM1_ESM.docx]

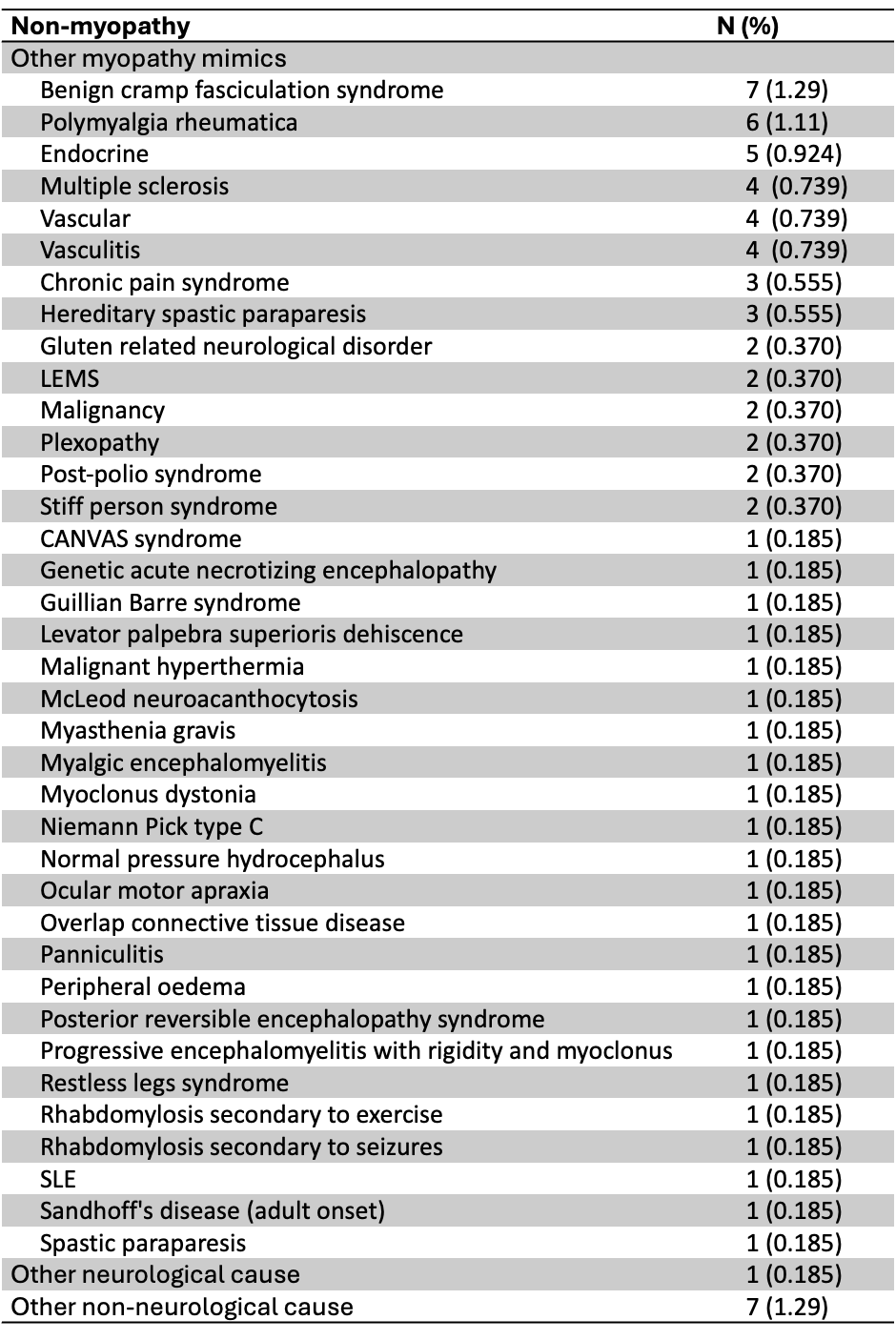


**Supplemental table 1. Additional non-myopathy diagnoses.**


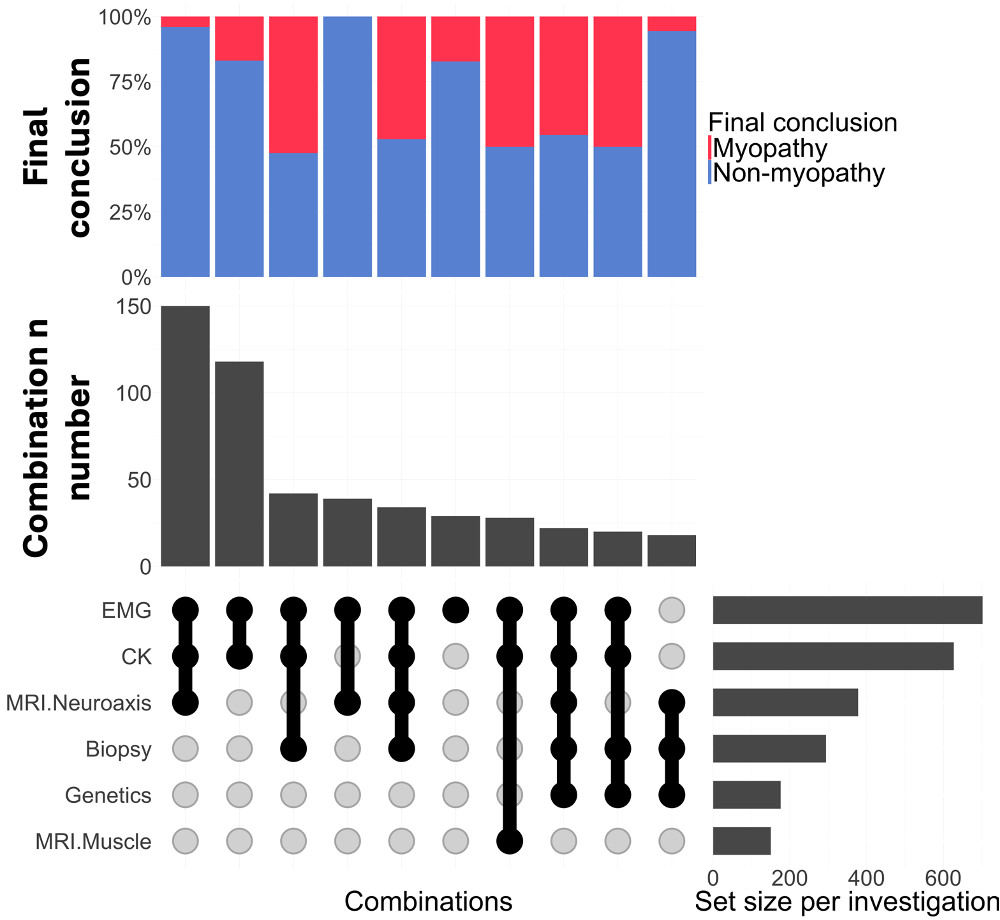


**Supplemental figure 1. Test combinations for the whole cohort.**

**
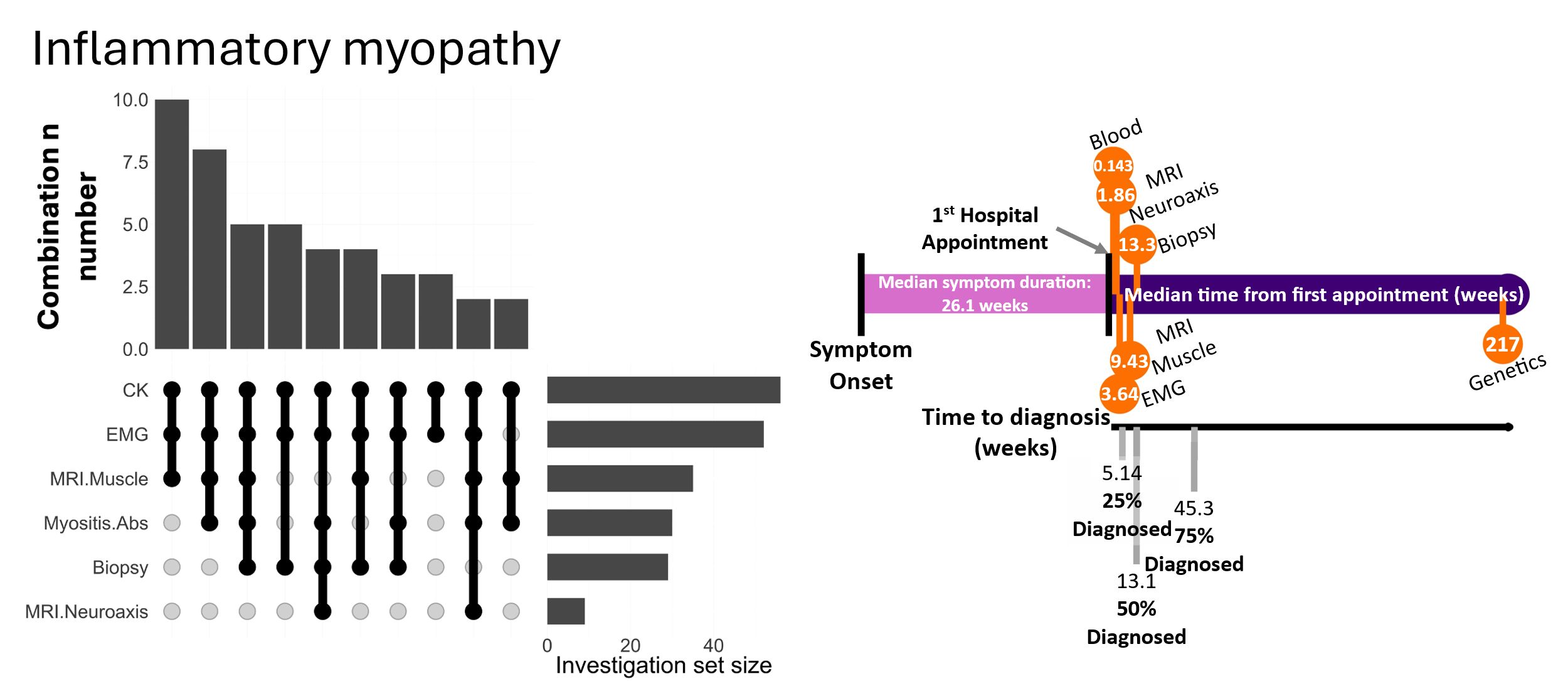
**

**Supplemental figure 2. Test combinations and overview timeline for inflammatory myopathy.**


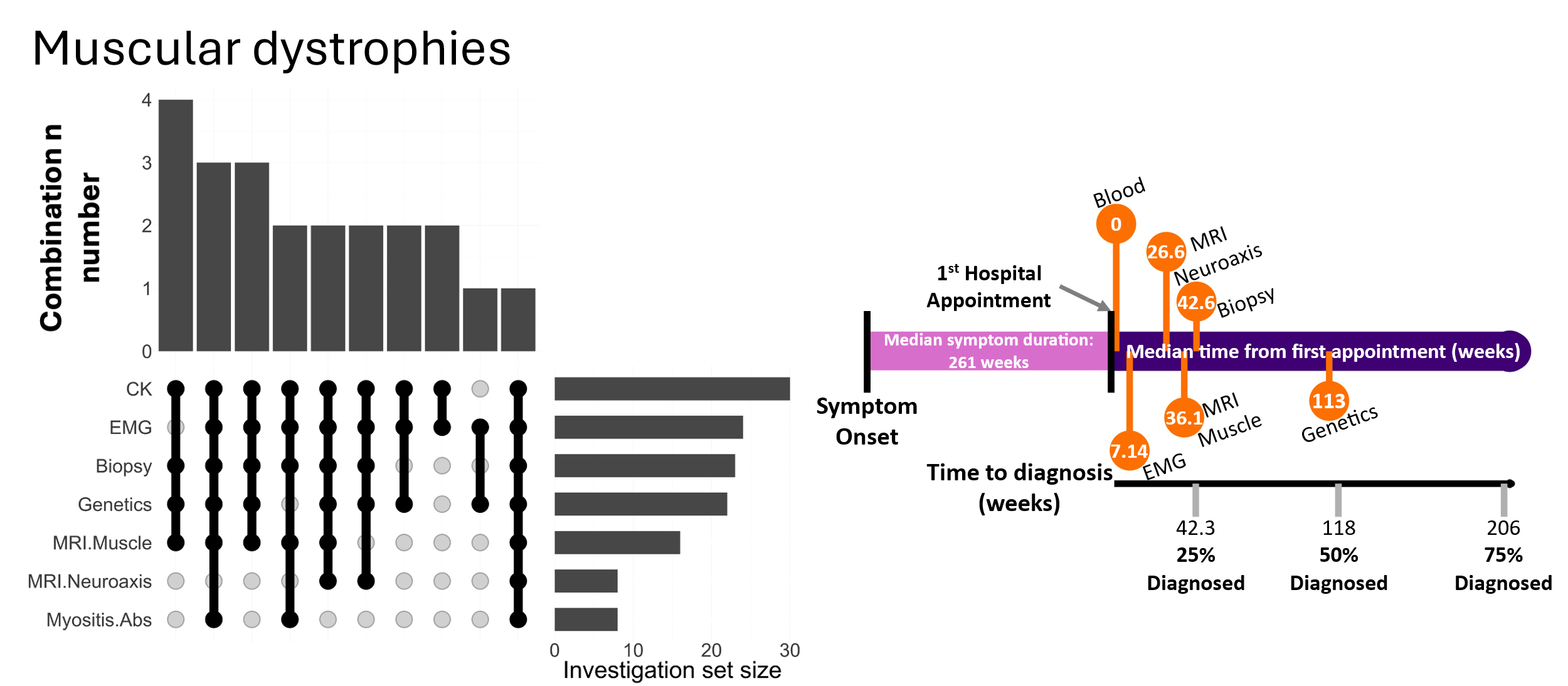


**Supplemental figure 3. Test combinations and overview timeline for muscular dystrophies.**


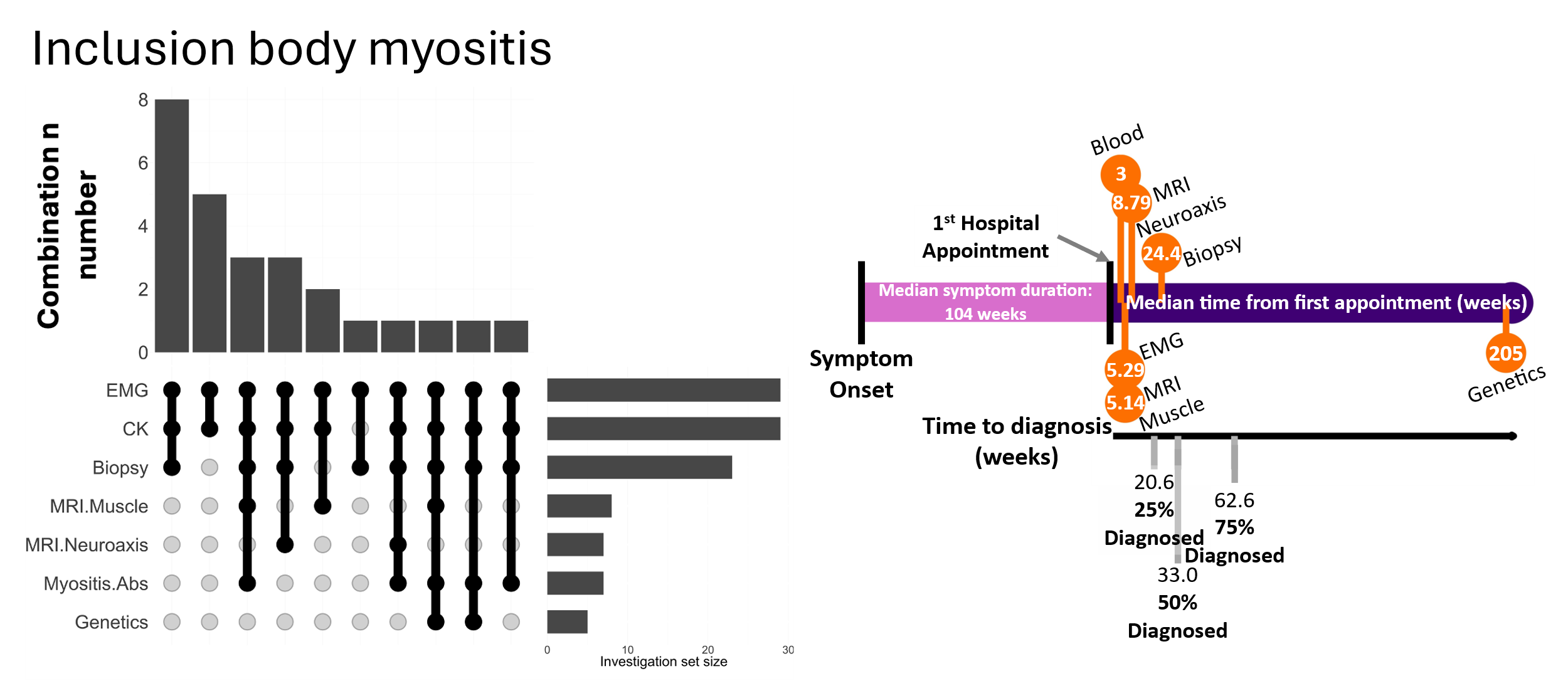


**Supplemental figure 4. Test combinations and overview timeline for inclusion body myositis dystrophies.**


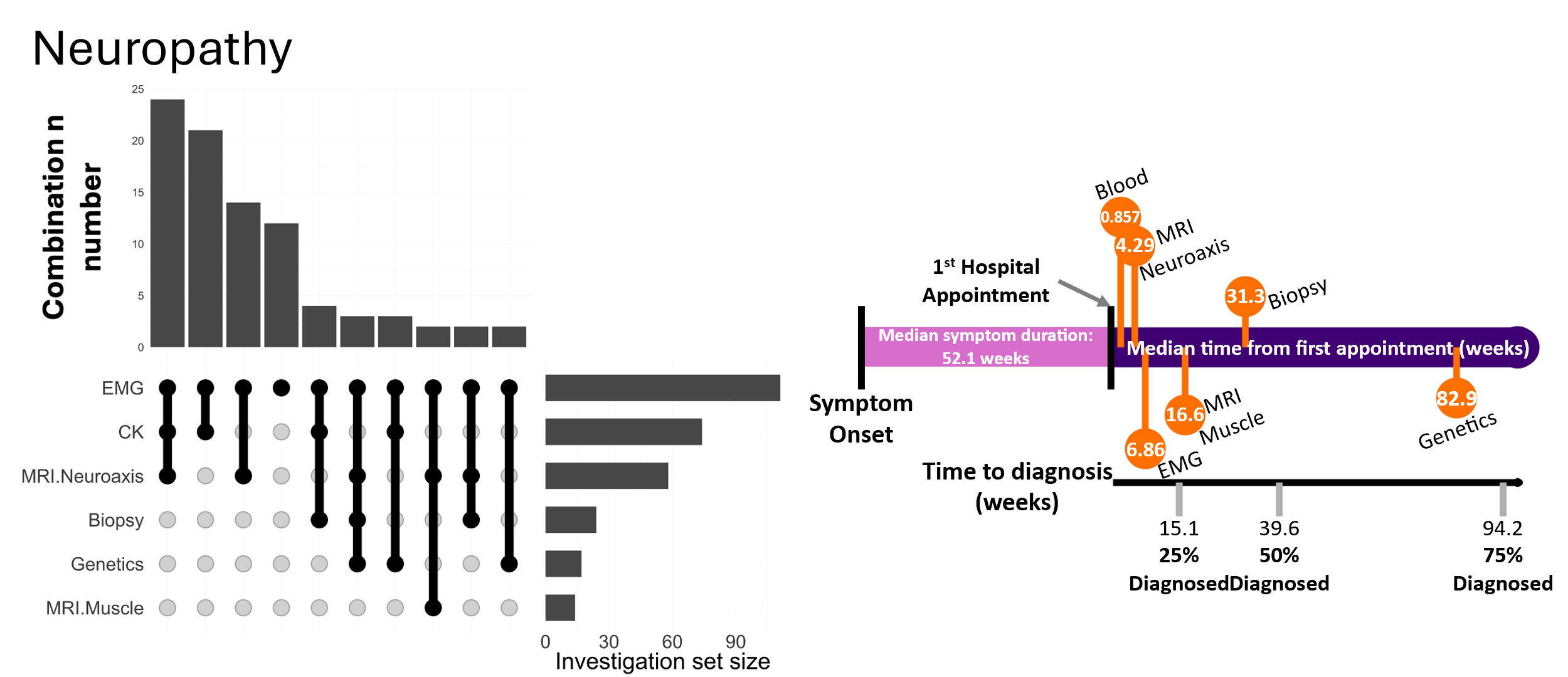


**Supplemental figure 5. Test combinations and overview timeline for the most common non-myopathy diagnosis: neuropathy.**


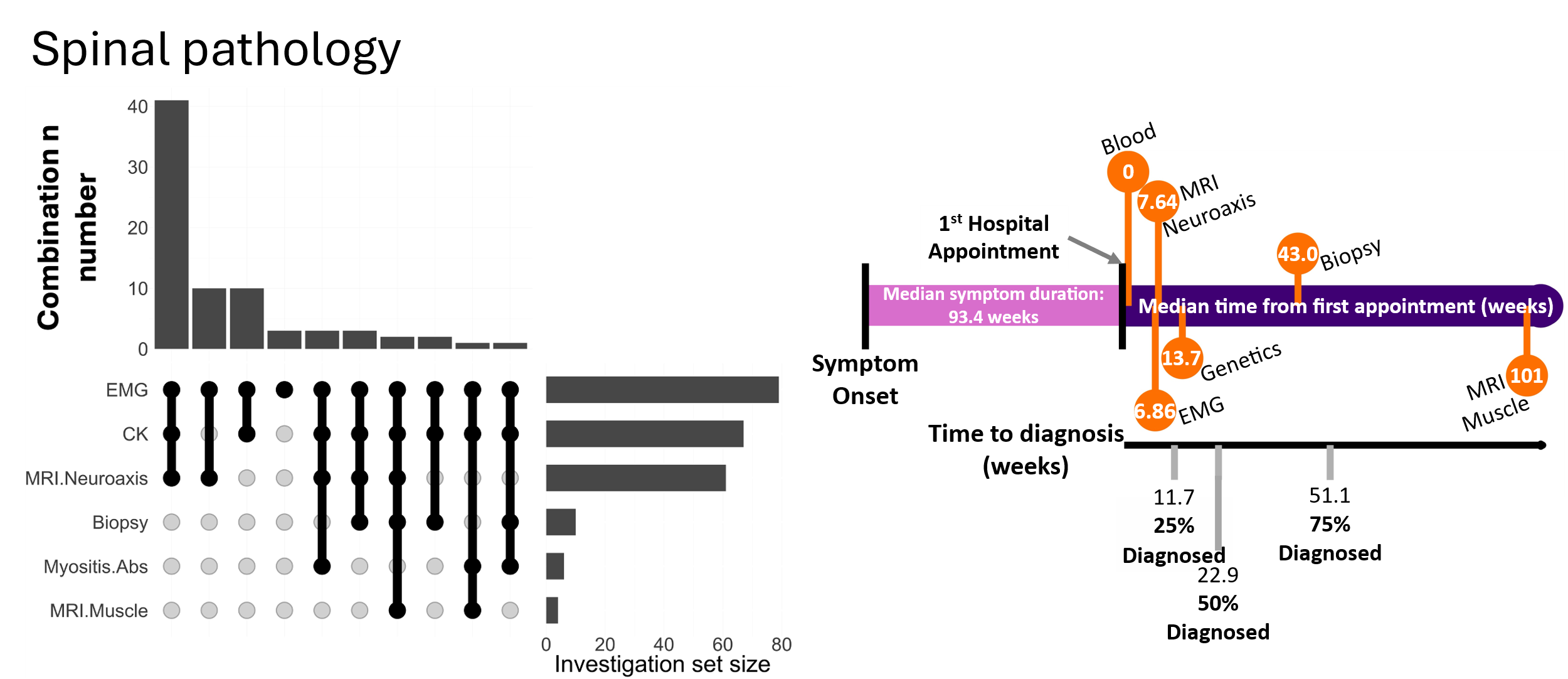


**Supplemental figure 6. Test combinations and overview timeline for the second most common non-myopathy diagnosis: spinal pathology.**


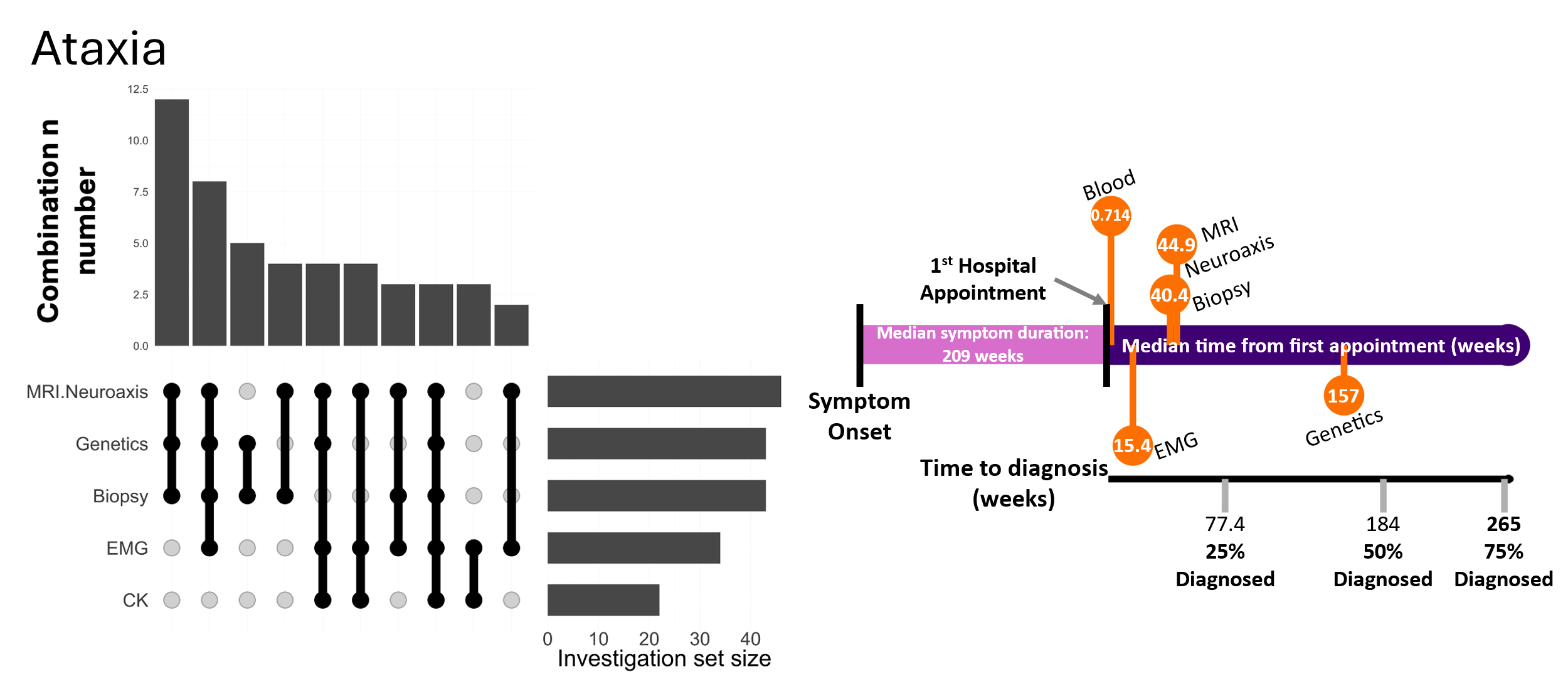


**Supplemental figure 7. Test combinations and overview timeline for the third most common non-myopathy diagnosis: ataxia.**


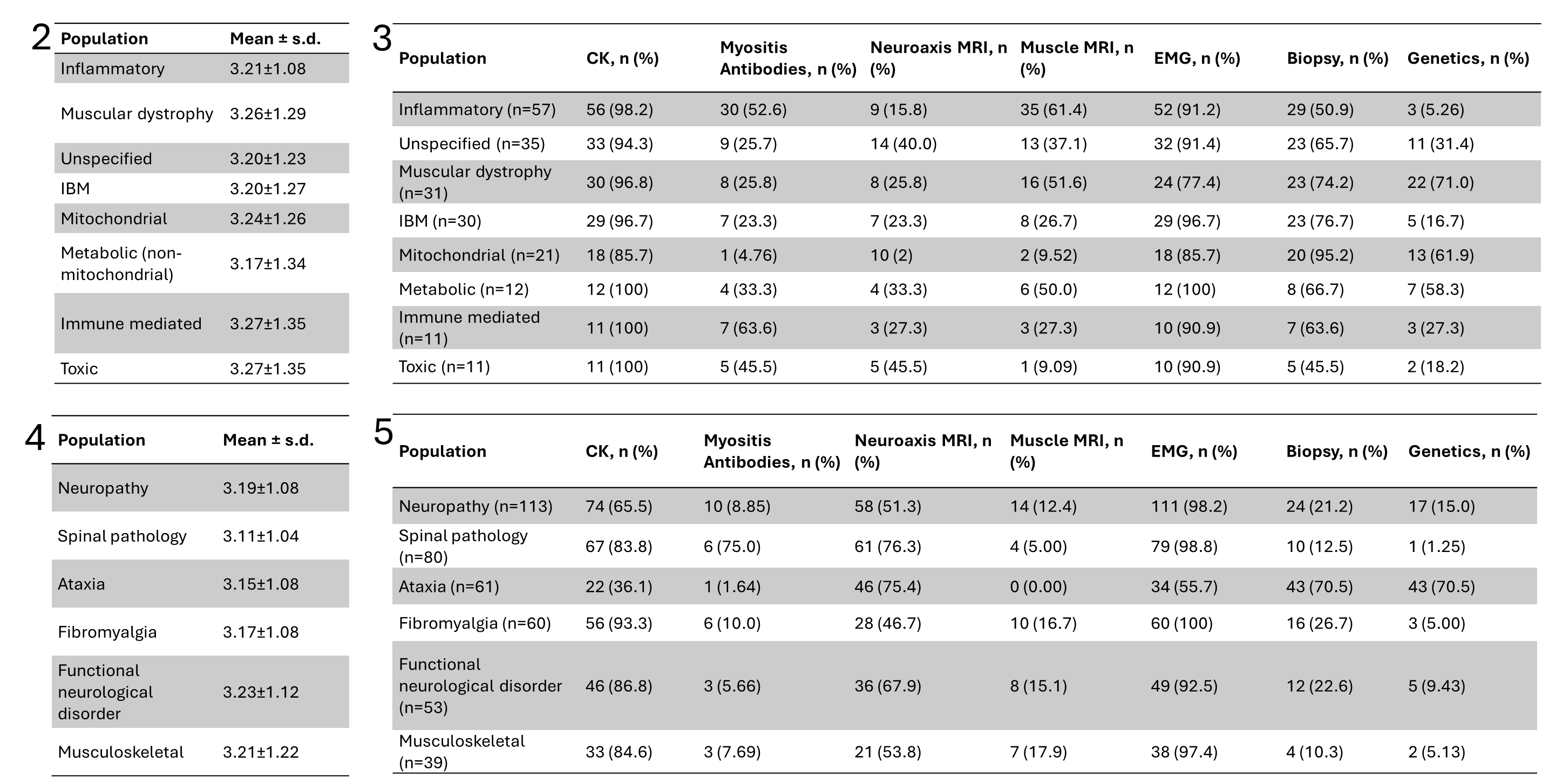


**Supplemental tables 2-5.**

2 & 3). Number of tests used in the myopathy subgroups (2) and utilisation of different tests (3).

4 & 5). Number of tests used in the 6 most common non-myopathy subgroups (4) and utilisation of different tests (5).


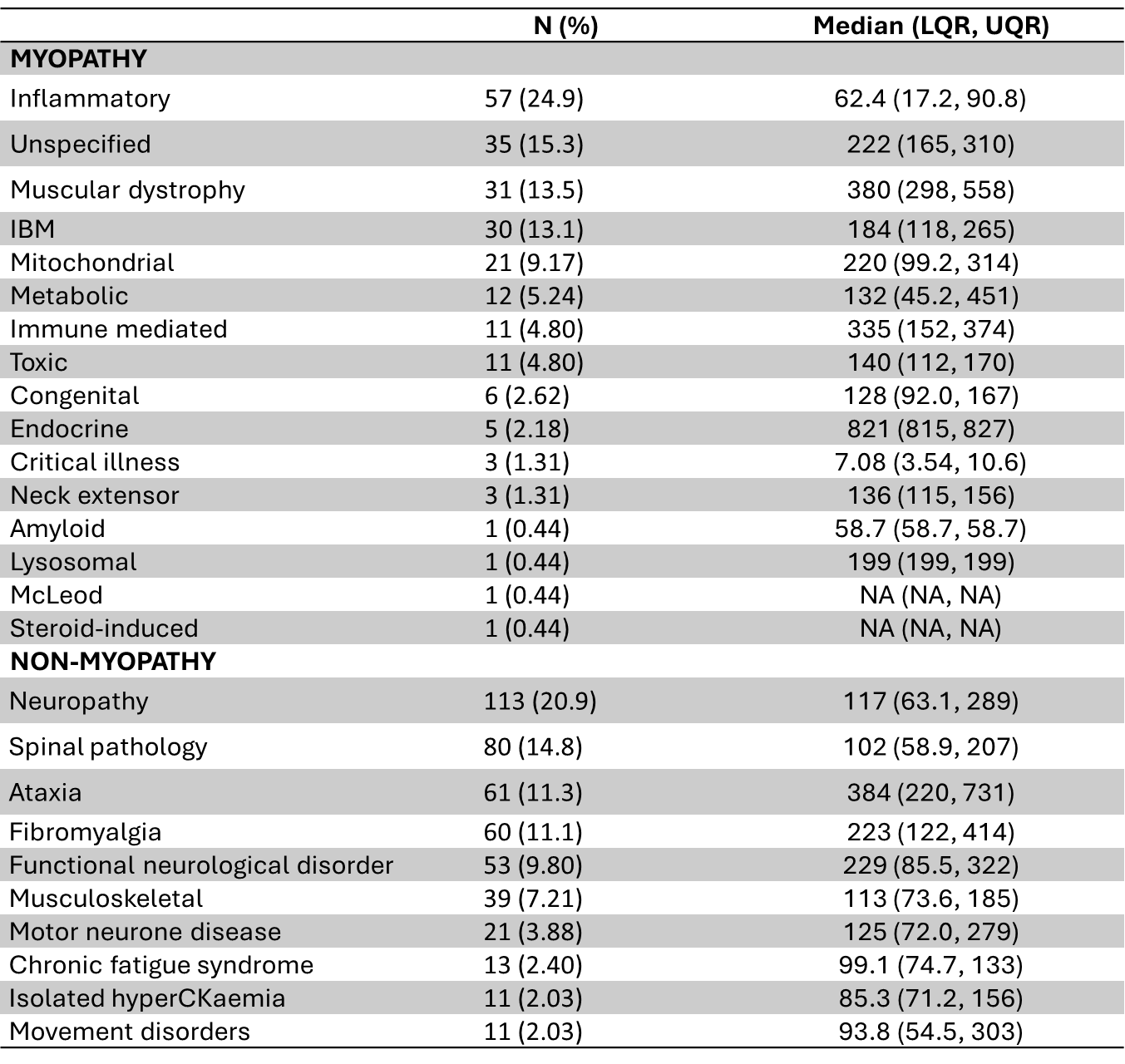


**Supplemental table 5. Time from symptom onset to diagnosis.**


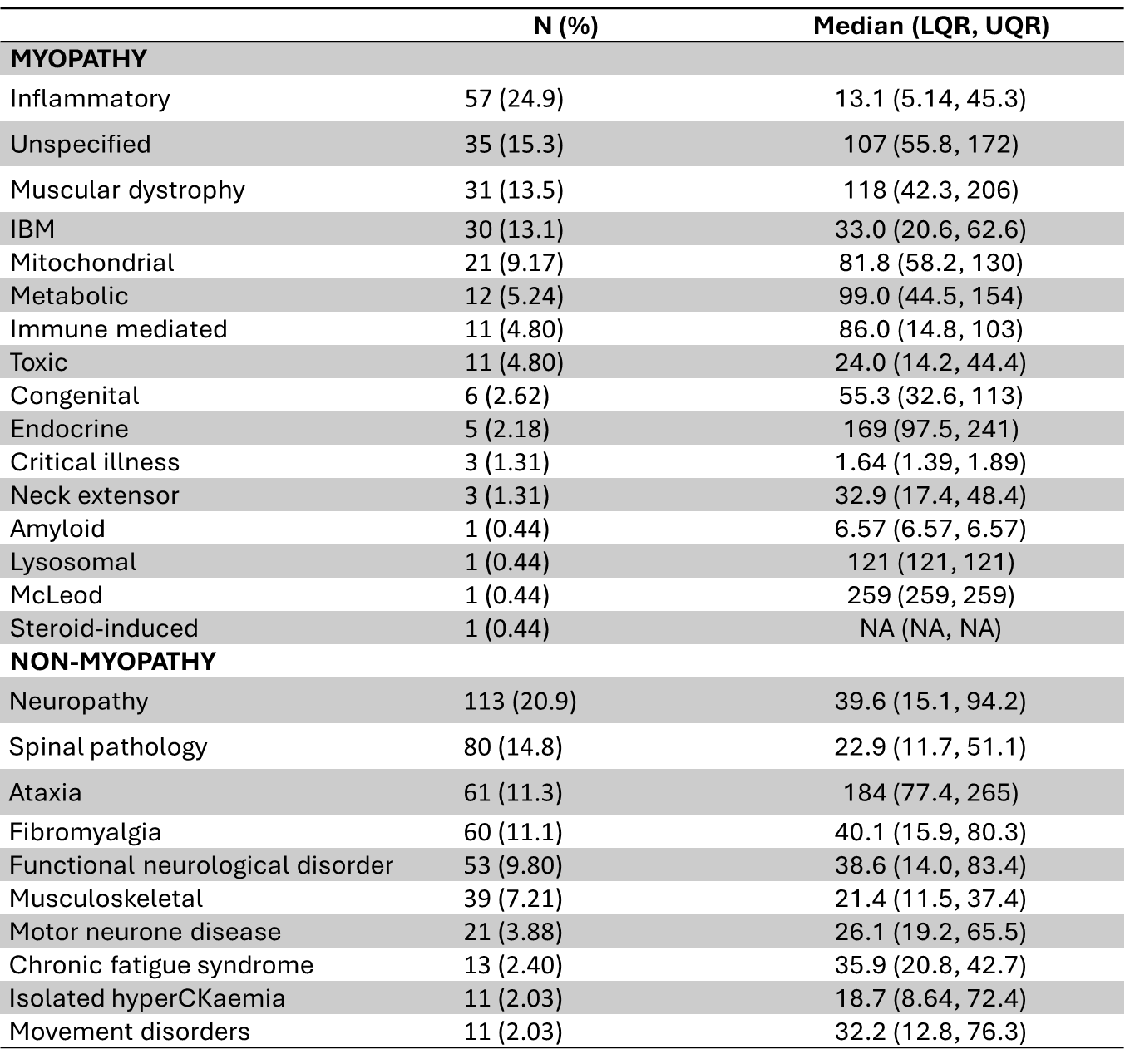


**Supplemental table 6. Time to diagnosis from 1^st^ hospital appointment.**


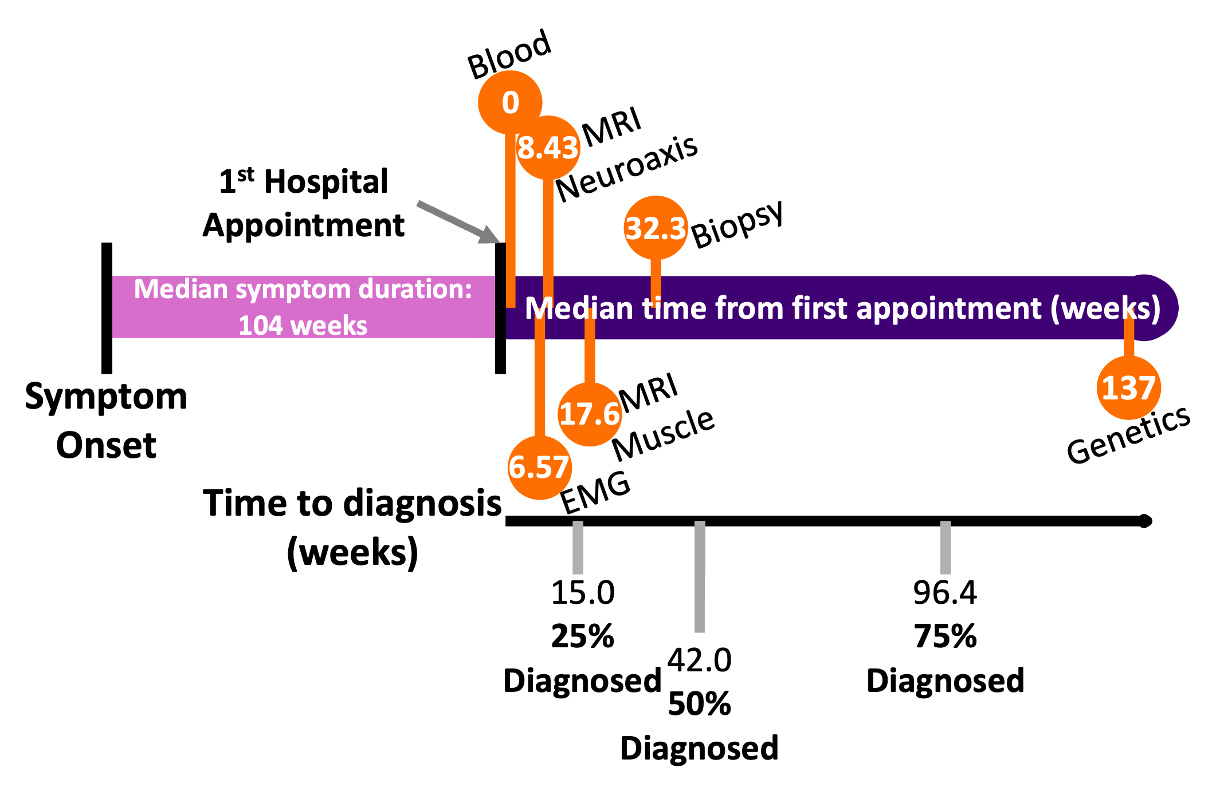


**Supplemental figure 8. An overview investigational and diagnostic timeline for the whole cohort.**
